# Coding of mechanical pain by myelinated and unmyelinated nociceptors in human hairy skin

**DOI:** 10.1097/PR9.0000000000001398

**Published:** 2026-01-30

**Authors:** Otmane Bouchatta, Oumie Thorell, Andrew G. Marshall, Merat Rezaei, Ahmed Barakat, Sarah McIntyre, Gregory J. Gerling, David A. Mahns, Håkan Olausson, Saad S. Nagi

**Affiliations:** a Department of Biomedical and Clinical Sciences, Center for Social and Affective Neuroscience, Linköping University, Linköping, Sweden; b School of Medicine, Western Sydney University, Australia; c Institute of Life Course and Medical Sciences, University of Liverpool, Liverpool, United Kingdom; d Department of Systems and Information Engineering, School of Engineering and Applied Science, University of Virginia, Charlottesville, VA, USA; e Department of Pharmacology and Toxicology, Faculty of Pharmacy, Assiut University, Assiut, Egypt

**Keywords:** A fiber, Mechanoreceptor, Microneurography, Neural coding, Nociceptor, Pain

## Abstract

**Introduction:**

In humans, cutaneous Aβ afferents are traditionally linked to discriminative touch, while pain is attributed to Aδ and C fibers. However, we previously identified thickly myelinated high-threshold mechanoreceptors (Aβ-HTMRs) that encode noxious skin indentations and evoke painful percepts when selectively activated. These afferents also display finely grained receptive fields and resilience to fatigue during sustained stimulation.

**Objectives:**

To characterize the tuning properties of A-HTMRs under controlled mechanical stimulation and compare them with C-HTMRs.

**Methods:**

We used a robotic stimulation system capable of delivering precise skin indentations across a wide force range (20–1000 mN). Single-unit axonal recordings (microneurography) were obtained from cutaneous afferents of the radial nerve in awake healthy participants. Both low- and high-threshold mechanoreceptors were recorded.

**Results:**

Among 39 recorded mechanoreceptive afferents, HTMRs were distinguished by high mechanical thresholds and lack of response to soft brushing, with conduction velocities in the Aβ- and C-fiber ranges. Both A- and C-HTMRs exhibited force-dependent increases in spike count and firing rate, with A-HTMRs showing significantly stronger responses. Principal component analysis revealed distinct separation between A- and C-HTMRs, driven by A-HTMRs' robust high-force responses. Psychophysical testing indicated painful stimuli were often described as “sharp,” and selective intraneural microstimulation of a single A-HTMR evoked localized “sharp-stinging” pain projected to its receptive field.

**Conclusion:**

Robot-controlled stimulation confirmed both A- and C-HTMRs' role in encoding painful mechanical stimuli. The fast conduction, high firing rates, fine receptive fields, and fatigue resilience of A-HTMRs suggest a specialized nociceptive system capable of conveying rich spatial-temporal information, potentially contributing to protective behaviors.

## Abstract 2

Supplemental Digital Content is Available in the Text.

Human myelinated nociceptors signal sharp mechanical pain rapidly and reliably.

## 1. Introduction

Pain is a complex, multidimensional experience that serves essential protective and adaptive functions. It encompasses sensory-discriminative, affective-motivational, and cognitive-evaluative components, each mediated by distinct but interacting neural systems.^1^ While the sensory dimension enables localization and encoding of noxious stimuli, the affective and cognitive dimensions shape the emotional valence and behavioral responses to pain. Despite significant advances in neuroscience, our understanding of how these dimensions are encoded and integrated remains incomplete, not least regarding the functional heterogeneity of nociceptors and the mechanisms underlying rapid pain signaling.

Traditional models in human physiology and medicine posit a dichotomy between touch and pain pathways: touch is conveyed by fast-conducting, thickly myelinated Aβ fibers, while pain is transmitted more slowly through thinly myelinated Aδ and unmyelinated C fibers.^15^ This framework implies that protective responses to painful stimuli should be slower than reactions to innocuous touch—a notion that seems counterintuitive given the evolutionary imperative for rapid detection and response to injury. Evidence from animal and human studies challenges this dichotomy. For instance, a subset of Aβ afferents in primate skin has been shown to function as nociceptors, comprising a substantial portion of the myelinated nociceptor population.^3,15^ Building on this foundation, we identified a distinct class of A afferents in humans that are insensitive to gentle touch, exhibit high mechanical thresholds, and encode noxious skin indentations.^6^ These fibers, which we term “ultrafast nociceptors,” conduct in the Aβ range and produce painful percepts when selectively activated through intraneural microstimulation (INMS; µA range). Their activation elicits sharp, stinging sensations, and individuals with selective Aβ deafferentation exhibit mechanical hypoalgesia, implicating these fibers in pain perception.^6^

In this study, we examined the electrophysiological response profiles of A- and C-high-threshold mechanoreceptors (HTMRs) using a robotic stimulator delivering highly precise mechanical stimuli. This approach enabled clear differentiation between afferent types, revealing that A- and C-HTMRs encode distinct features of noxious mechanical input. Together, our findings underscore the functional diversity of nociceptors and offer new insights into the peripheral mechanisms of mechanical pain in humans.

## 2. Methods

### 2.1. Participants

Thirty-seven healthy participants (18–40 years) were recruited through university and social media advertisements. The exclusion criteria for this study comprised neurological or musculoskeletal disorders, skin diseases (eg, psoriasis), diabetes, and recent use of pain-relieving or psychoactive medication. All participants provided informed written consent before the start of the experiment. The study was approved by the Swedish Ethical Review Authority (Dnr 2015/305-31; Dnr 2020-04207).

Participants were seated in a chair with legs or arms extended and supported using vacuum pillows—with the hand pronated for radial-nerve recordings. Care was taken to ensure that each participant was comfortably seated and acclimated to the room temperature (22°C) before starting the experiment. If participants reported feeling cold, a blanket was provided, leaving only the test region exposed.

### 2.2. Microneurography

Single-unit axonal recordings (microneurography) were obtained from the cutaneous fascicles of the radial nerve in 16 participants (11 men, 5 women). Under real-time ultrasound guidance (LOGIQ P9, GE Healthcare, Chicago, IL), the radial nerve was impaled with a Tungsten microelectrode (FHC Inc, Bowdoin, ME). An uninsulated reference electrode was inserted adjacent to the recording electrode, just under the skin. A high-impedance preamplifier (MLT185 headstage, ADInstruments, Sydney, Australia) was attached to the skin near the recording electrode and used together with a low-noise high-gain amplifier (FE185 Neuro Amp EX, ADInstruments).

All data presented in this study were obtained from new experiments. Single units were searched for by applying soft and rough brushing, as well as skin pinching. The soft brush was composed of goat hairs and the coarse brush of rigid synthetic fibers; both measured 5 cm in width. Mechanical thresholds were determined using Semmes‐Weinstein monofilaments (nylon fiber; Aesthesio, Bioseb, Pinellas Park, FL). All recorded afferents were mechanically responsive and classified into subtypes based on established criteria.^6,18^ Receptive fields were confirmed to be cutaneous by verifying movement with skin translation and response to mechanical probing during skin lifting (pinch).

Conduction velocity of the recorded afferent was estimated from latency responses to surface electrical stimulation of the receptive field (FE180 Stimulus Isolator, ADInstruments). Electrically evoked spikes were detected using a spike-detection algorithm and independently verified by two trained observers based on consistent latency, waveform morphology, and inspection on an expanded time scale, confirming that they originated from the same unit. To assess force coding, individual HTMRs were tested using a robotic indenter (Aurora Scientific, model 305C-I, Ontario, Canada) with a 1-mm flat tip indenting the receptive field (0.2 seconds). The indenter was mounted on an X-Y plotter to improve maneuverability during microneurography. Each trial began at skin contact and ended on withdrawal. Five target forces—20, 60, 100, 260, and 1000 mN—were delivered in pseudo-random order, with each level repeated at least 3 times per afferent, except in one unit where each force was tested once. Successful recordings using robotic stimulation were obtained from 3 A-HTMRs and 3 C-HTMRs, each from a different participant.

Neural activity was sampled at 20 kHz and recorded using LabChart software (v8.1.24, ADInstruments) and PowerLab hardware (6/35, PL3516/P). Data were exported to Spike2 (v10.13, Cambridge Electronic Design Ltd., Cambridge, United Kingdom) for offline analysis. Recorded action potentials were carefully examined on an expanded time scale. Threshold crossing was used to distinguish action potentials from noise with a signal-to-noise ratio of at least 2:1, and spike morphology was confirmed by template matching. Spike sorting used a template-matching approach, suitable for discriminating A-fiber activity given their high signal-to-noise ratio and consistent waveform morphology. For C fibers, we used strict validation criteria to ensure single-unit isolation. Spike clusters were manually inspected for consistent waveform shape and firing patterns, cross-correlation was used to identify potential overlap between units, and receptive-field mapping confirmed that recorded activity corresponded to the same fiber's responses. Recordings were discarded if multiple units were present or if spike amplitudes were not distinct from the noise, preventing secure action potential identification.

When time permitted, weak electrical impulses (0.2 ms, 1 Hz, µA range) were delivered through the recording electrode, starting from 0 µA and increasing in small increments until a sensation was reported. To determine whether the electrically evoked percept originated from the recorded afferent, we assessed spatial overlap between the projected field (evoked percept) and the physiologically mapped receptive field.^8,19^ Participants were asked to report the location of the perceived sensation relative to the site of mechanical stimulation.^10^ When receptive field–projected field overlap was confirmed, participants were asked to describe the quality of the electrically evoked sensation. Their responses were supplemented using a structured questionnaire (modified from Vallbo et al.^19^).

### 2.3. Psychophysics

Participants were seated comfortably with the dorsum of the hand stabilized. Psychophysical responses were collected from 21 healthy participants (9 men and 12 women) in response to the same 5 force levels (20, 60, 100, 260, and 1000 mN) delivered by the Aurora indenter, as described above. Each force level was applied 3 times in a randomized order. After each stimulus, participants rated the unpleasantness of the sensation using a visual analog scale (VAS; 0 = neutral to 10 = very unpleasant).

On completing all trials, participants filled out the McGill pain questionnaire to characterize the quality of sensations evoked by indentation forces. Participants could select any number of descriptors and ranked each as mild, moderate, or severe.

### 2.4. Data analysis

Data are presented as individual and mean (±SEM) responses. To account for differences in how participants used the VAS, unpleasantness ratings were binarized (VAS > 0 = unpleasant) and expressed as incidence—the proportion of trials at each force level in which any unpleasantness was reported. Statistical analyses were conducted using GraphPad Prism (version 9.1.2, GraphPad Software Inc, La Jolla, CA). The specific statistical tests used are reported alongside the relevant results. Principal component analysis (PCA) was used to visualize relationships between neuronal units and their electrophysiological response variables. The data were analyzed twice: first, using units as a grouping variable, with the 3 response metrics (number of spikes, mean frequency, and peak frequency) across indentation forces. Second, using both unit type and indentation forces as grouping variables, and the 3 metrics as response variables. This 2-level approach enabled visualization of variability associated with both unit type and stimulus intensity. Missing values were imputed using a custom function that replaced missing entries with the mean response of other units of the same type at the same indentation force. This imputation was performed separately for each neural metric, preserving unit type-specific patterns while minimizing data loss. To quantify the heterogeneity of unit type across indentation forces, we calculated the mean Euclidean distance to the centroid of each group in the PCA space. For each unit type and force level, the centroid was defined as the mean coordinate along the first 2 principal components (PC1, PC2). The mean distance to centroid (and its standard deviation) for each group provided a measure of within-group dispersion, representing the degree of heterogeneity in neuronal responses. Data wrangling, analysis, and visualization for the PCA were performed in R programming language (version 4.5.1) using the packages tidyverse (v 2.0.0) for data wrangling, FactoMineR (v 2.12) for PCA analysis, factoextra (v 1.07) for PCA visualization, missMDA (v 1.20) for handling the missing data, and ggplot2 (v 3.5.2) for data visualization.

## 3. Results

### 3.1. Sample composition and unit classification

**Figure 1.**
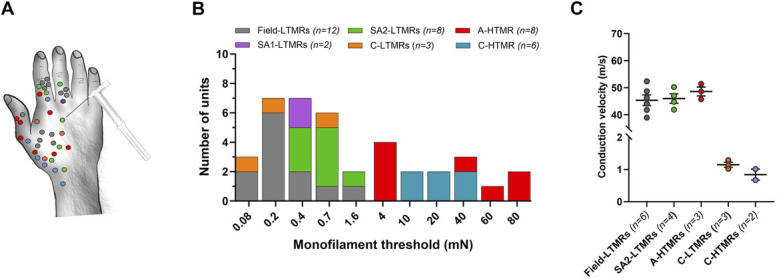
Characteristics of the recorded afferents. (A) Receptive field locations of all units recorded from the radial nerve. (B) Mechanical threshold distribution of LTMRs and HTMRs. LTMRs had mechanical thresholds ≤1.6 mN, while HTMRs had mechanical thresholds ≥ 4 mN. (C). Conduction velocities based on surface electrical stimulation. The data show individual and mean (±SEM) velocities. A-HTMRs had conduction velocities similar to A-LTMRs (A-HTMRs: 48.5 ± 1.6 m/second; field-LTMRs: 45.3 ± 1.9 m/second; SA2-LTMRs: 45.9 ± 1.7 m/second; F_(2,10)_ = 0.63; *P* = 0.5509, one-way ANOVA). C-LTMRs and C-HTMRs had velocities of 1.1 ± 0.1 m/second and 0.8 ± 0.2 m/second, respectively. ANOVA, analysis of variance; HTMR, high-threshold mechanoreceptor; LTMR, low-threshold mechanoreceptor.

**Figure 2.**
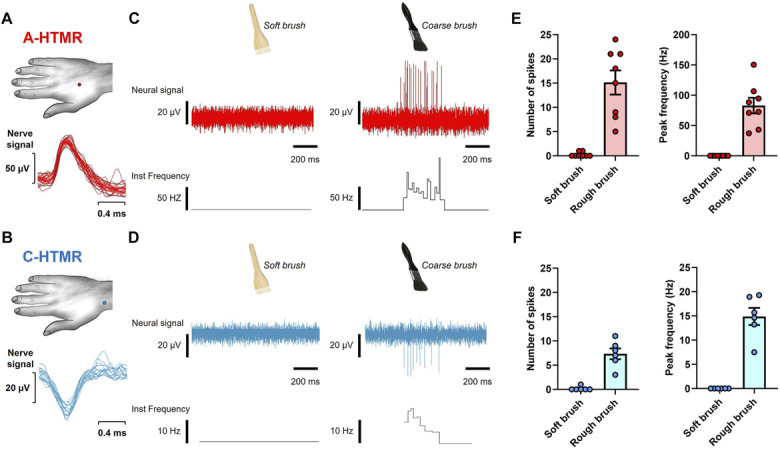
Brush responses of A- and C-HTMRs. (A and B) Receptive field locations of representative A- and C-HTMRs, with superimposed spike activity in response to brushing the receptive field. (C and D) Example traces showing neural responses and instantaneous firing frequencies to soft and coarse brush strokes. (E and F) Spike counts and peak firing frequencies of A-HTMRs (n = 8) and C-HTMRs (n = 6) in response to brush stroking at approximately 3 cm/second. Data are shown as individual and mean (±SEM) responses. HTMR, high-threshold mechanoreceptor.

We recorded 39 units from the radial nerve comprising 6 afferent types: field-low-threshold mechanoreceptor (LTMR) (n = 12), SA1-LTMR (n = 2), SA2-LTMR (n = 8), C-LTMRs (n = 3), A-HTMRs (n = 8), and C-HTMRs (n = 6). The receptive field locations of all recorded afferents are shown in Figure 1A. All LTMRs displayed low indentation thresholds (≤1.6 mN), whereas all HTMRs had high indentation thresholds (≥4 mN; Fig. 1B). Conduction velocities, where measured (field-LTMR [n = 6], SA2-LTMR [n = 4], C-LTMRs [n = 3], A-HTMRs [n = 3], and C-HTMRs [n = 2]), were within the Aβ range (>30 m/second) for all A fibers and around 1 m/s for C fibers (Fig. 1C). A- and C-HTMRs were unresponsive to a soft brush but responded to a coarse brush (Fig. 2).

### 3.2. A-high-threshold mechanoreceptors preferentially encode mechanical pain

**Figure 3.**
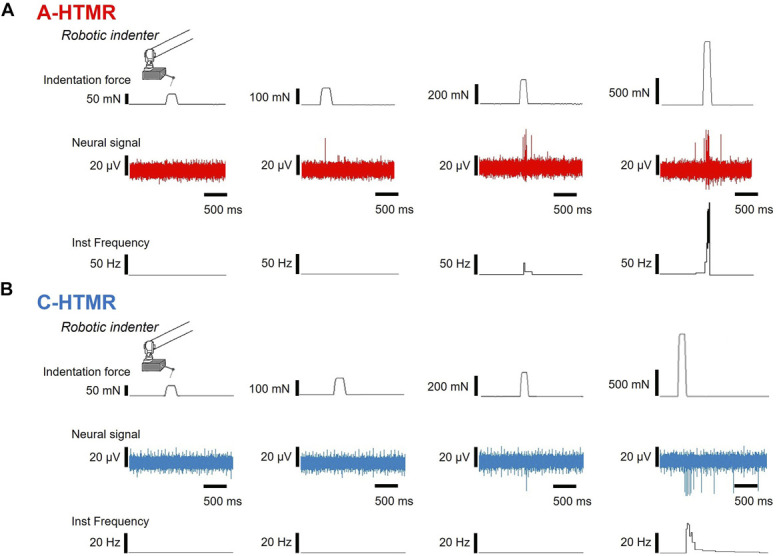
Examples of neural responses to indentations. Traces show indentation force markers, raw neural signals, and instantaneous frequency for representative A- and C-HTMR units in response to 60, 100, 260, and 1000 mN forces. HTMR, high-threshold mechanoreceptor.

**Figure 4.**
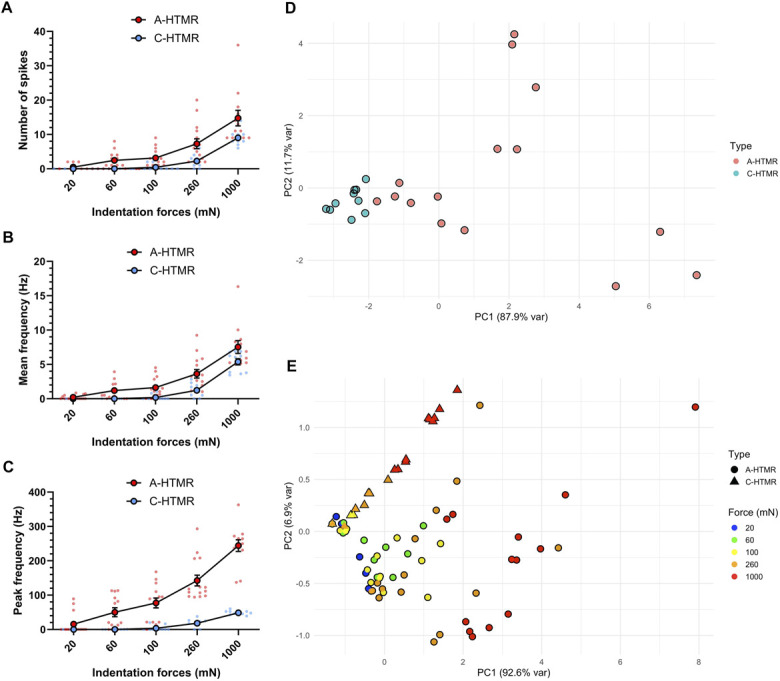
A- and C-HTMRs encode painful skin indentation. (A–C) Number of spikes, mean frequency, and peak frequency of A-HTMRs (n = 3) and C-HTMRs (n = 3) across 5 indentation forces delivered using a robotic indenter. Values shown as individual and mean ± SEM. For A-HTMRs, indentation force had a significant effect on spike count (F_(5,77)_ = 23.49, *P* < 0.001), mean frequency (F_(5,77)_ = 32.26, *P* < 0.001), and peak frequencies (F_(5,77)_ = 49.74, *P* < 0.001; one-way ANOVA). To illustrate this, we compared peak firing rates at 1000 mN with those at all lower forces. Tukey post hoc tests confirmed that responses at 1000 mN were significantly higher than each of the lower force levels ([20 vs 1000]: q = 17.86, *P* < 0.001; [60 vs 1000]: q = 14.65, *P* < 0.0001; [100 vs 1000]: q = 12.85, *P* < 0.0001; [260 vs 1000]: q = 7.94, *P* < 0.0001). For C-HTMRs, indentation force also significantly affected spike count (F_(5,39)_ = 54.60, *P* < 0.001), mean frequency (F_(5,39)_ = 53.63, *P* < 0.001), and peak frequencies (F_(5,39_) = 62.14, *P* < 0.001, one-way ANOVA). Peak firing rates at 1000 mN were significantly greater than all lower forces ([60 vs 1000]: q = 18.27, *P* < 0.0001; [100 vs 1000]: q = 19.51, *P* < 0.0001; [260 vs 1000]: q = 12.42, *P* < 0.0001; Tukey multiple comparisons test). (D) PCA plot of neural activity metrics—including spike count, mean firing rate, and peak firing frequency—for A- and C-HTMRs across varying force levels, using unit type as a grouping variable. (E) PCA plot of neural activity metrics of A- and C-HTMRs, using unit type and indentation force as grouping variables. ANOVA, analysis of variance; HTMR, high-threshold mechanoreceptor; PCA, principal component analysis.

To quantify force-coding, we applied 5 indentation forces (20, 60, 100, 260, and 1000 mN), with at least 3 repeats per unit (Figs. 3 and 4). Both A- and C-HTMRs fired monotonically with force, displaying a linear stimulus–response relationship (A-HTMRs: slope = 0.22, 95% CI 0.11–0.33, F_(1,4)_ = 30.69; R^2^ = 0.88, *P* = 0.0052; C-HTMRs: slope = 0.04, 95% CI 0.03–0.06, F_(1,4)_ = 179.5; R^2^ = 0.97, *P* = 0.0002). Post hoc comparisons focused on 1000 mN vs all lower forces—an approach informed by our earlier finding that field-type tactile afferents plateau once forces become painful,^4^ whereas HTMRs continue to scale (see Fig. 4 legend for statistics). Across all neural measures—spike count, mean frequency, and peak frequency—A-HTMRs responded significantly more vigorously than C-HTMRs (spike count F_(1,23_ = 10.95, *P* = 0.0031; mean frequency F_(1,23)_ = 10.18, *P* = 0.0041; peak frequency F_(1,23)_ = 45.97, *P* < 0.0001; 2-way mixed analysis of variance with Tukey-corrected post hoc tests; Figs. 4A–C). Principal component analysis, using unit type as a grouping variable, revealed a clear distinction between A-HTMRs and C-HTMRs (Fig. 4D). When both unit type and indentation force were considered, PCA showed that A-HTMRs responded more robustly overall, particularly to high-force stimuli. By contrast, C-HTMRs exhibited lower overall activity but showed relatively enhanced responsiveness at higher force levels (Fig. 4E). These findings suggest that although C-HTMRs are preferentially activated by stronger mechanical stimuli, their response magnitude remains lower than that of A-HTMRs. Centroid distance analysis further indicated that increasing indentation force leads to greater heterogeneity in both A- and C-HTMR populations (Supplementary Fig. 1, http://links.lww.com/PR9/A376), with A-HTMRs displaying a higher degree of heterogeneity compared with C-HTMRs.

**Figure 5.**
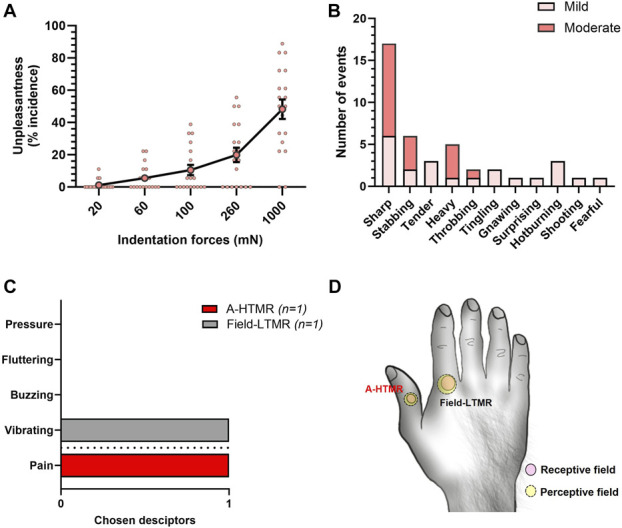
Incidence and quality of unpleasantness in response to Aurora mechanical stimulation. (A) Individual and mean (±SEM) unpleasantness incidence (21 participants) across 5 force levels (20, 60, 100, 260, 1000 mN). The incidence of unpleasantness increased with increasing forces. (B) Sensory quality of Aurora-induced percepts, based on the McGill pain questionnaire. “Sharp” and “stabbing” were the most frequently reported descriptors. (D) Microstimulation of a single field-LTMR and an A-HTMR elicited distinct sensory percepts. (E) Locations of the electrically evoked perceptive fields correspond to the physiologically mapped receptive fields. The sizes of receptive and perceptive field locations are shown schematically to illustrate the spatial overlap required to confirm successful single-unit activation. HTMR, high-threshold mechanoreceptor; LTMR, low-threshold mechanoreceptor.

In psychophysical testing, unpleasantness incidence increased with force (Fig. 5A). On the McGill pain questionnaire, “sharp” was the most frequently chosen descriptor, followed by “stabbing” (Fig. 5B). Intraneural microstimulation of one A-HTMR produced a well-localized (≤1 cm) “sharp-stinging” pain that colocalized with the physiologically mapped receptive field (Figs. 5C and D). The unit's conduction velocity was 48.6 m/second. Its receptive field contained several high-sensitivity spots, including a hotspot with a slowly adapting discharge profile. Intraneural microstimulation was delivered using an ascending–descending series, with 9 µA (1 Hz, 0.2 ms) as the perceptual threshold. During ongoing 1-Hz stimulation, participants reported that the pain sensation intensified over time. By contrast, INMS of Aβ-LTMRs produces nonpainful tactile sensations—such as vibration, flutter, or pressure—depending on fiber type and stimulation parameters, as previously shown.^6,9,19^ For example, INMS of one field-LTMR produced a “vibration” sensation at 6 µA (30 Hz, 0.2 ms)—projected to the unit's receptive field—with higher stimulation frequencies (60 and 120 Hz) resulting in a graded increase in perceived intensity (Figs. 5C and D).

## 4. Discussion

Nociception is a multifaceted system encompassing diverse neuronal populations, sensitivity profiles, propensity for plastic change, and tissue-specific specializations, among other factors.^1^ In this study, we used a robotic indenter to deliver well-controlled indentation forces and demonstrated that A-HTMRs in human hairy skin fulfill classical nociceptor criteria: high mechanical thresholds and the ability to encode painful stimuli.^4^ In addition to being well-controlled, the indentation trajectories were verifiable with a force sensor built into the indenter, and the tip contacting the skin was identical for all forces. These features stand in contrast to the Semmes Weinstein monofilaments used in earlier studies,^6^ which are delivered by hand, are not measured, and have differently sized tips for different forces. Moreover, conduction velocities of A-HTMRs overlapped with those of Aβ-LTMRs, indicating that mechanical pain can be signaled as rapidly as discriminative touch—an advantage for fast, protective reflexes.^13,20^ These findings extend earlier evidence for ultrafast nociceptors and align with recent transcriptomic studies identifying A-HTMRs in human dorsal root ganglia.^6,12,21^ We recently identified a subtype of A-HTMRs responsive to cooling but not heating, corresponding to the human Peptidergic (hPEP).KIT population in human Dorsal Root Ganglia (DRG) single-soma transcriptomics, characterized by the expression of KIT (as the name implies), Transient Receptor Potential Melastatin 8 (TRPM8), and Piezo-type mechanosensitive ion channel component 2 (PIEZO2) and by the absence of Transient Receptor Potential Vanilloid 1 (TRPV1).^2,21^ In this study, we focused on force coding and did not differentiate between cool-sensitive and -insensitive A-HTMRs, as both respond similarly to skin indentation.^2,21^

Principal component analysis of electrophysiological data confirmed distinct functional groups for A- and C-HTMRs, with notable differences in force-coding dynamics and response variability. C-HTMRs displayed relatively uniform responses, whereas A-HTMRs showed greater heterogeneity across indentation forces. Although the functional implication of this remains uncertain, the data suggest that A-HTMRs may operate over a broader range of forces, compared with C-HTMRs. Their high firing rates, resistance to fatigue, and precise receptive fields make them well-suited for encoding mechanical pain.^6,7^ In addition, their fast conduction velocity enables rapid withdrawal from harmful stimuli,^13,14^ whereas C-fibers may support longer-term behavioral adaptations.

Analyzing spike patterns in C-fibers using microneurography remains challenging due to the constraints of single-electrode recordings and the high similarity of spike waveforms across units. These factors highlight the need for ground-truth validation and optimized recording configurations to improve the reliability of C-fiber data interpretation.^16^

Intraneural microstimulation of individual A-HTMRs evoked a sharply localized “sharp-stinging” pain, whereas INMS of Aβ-LTMRs yields nonpainful tactile sensations. Clinical evidence further supports the role of Aβ nociceptors in human pain perception. Two rare individuals with selective Aβ deafferentation exhibited reduced mechanical pain sensitivity despite preserved Aδ and C-fiber function.^6^ This observation challenges the prevailing view that mechanical pain is exclusively mediated by the Aδ system. Indeed, current quantitative sensory-testing protocols use cold detection and mechanical pain testing for assessment of the Aδ system.^11^ However, in Aβ-deafferented individuals, cold perception was intact while punctate mechanical pain was impaired—highlighting the contribution of Aβ-HTMRs.^6^ While our study focuses on the role of A-HTMRs in acute mechanical nociception, their potential involvement in chronic pain states is not yet understood and is the subject of ongoing work.

In conclusion, our data confirm a population of fast nociceptors in human skin that encode painful mechanical stimuli and conduct at Aβ speeds. These findings add to evidence that challenges the long-standing dichotomy between fast-conducting touch afferents and slow-conducting nociceptors. The molecular identification of these myelinated nociceptors opens new avenues for understanding pain mechanisms and developing targeted therapies.

## Disclosures

The authors have no conflict of interest to declare.

## Supplemental digital content

Supplemental digital content associated with this article can be found online at http://links.lww.com/PR9/A376.

## Supplementary Material

SUPPLEMENTARY MATERIAL
