# Supplementary material for: Coding of mechanical pain by myelinated and unmyelinated nociceptors in human hairy skin
Source: Pain Rep. 2026 Jan 30;11(2):e1398. doi: 10.1097/PR9.0000000000001398 (PMC12863872; doi:10.1097/PR9.0000000000001398)
Supplement: SUPPLEMENTARY MATERIAL [file painreports-11-e1398-s001.pdf]

Coding of Mechanical Pain by Myelinated and Unmyelinated Nociceptors in Human Hairy Skin

Otmane Bouchatta[a,*], Oumie Thorell[a,b], Andrew G. Marshall[c], Merat Rezaei[d], Ahmed Barakat[a,e], Sarah McIntyre[a], Gregory J. Gerling[d], David A. Mahns[b], Håkan Olausson[a], Saad S. Nagi[a,b].

Supplementary material contains supplementary figure 1.

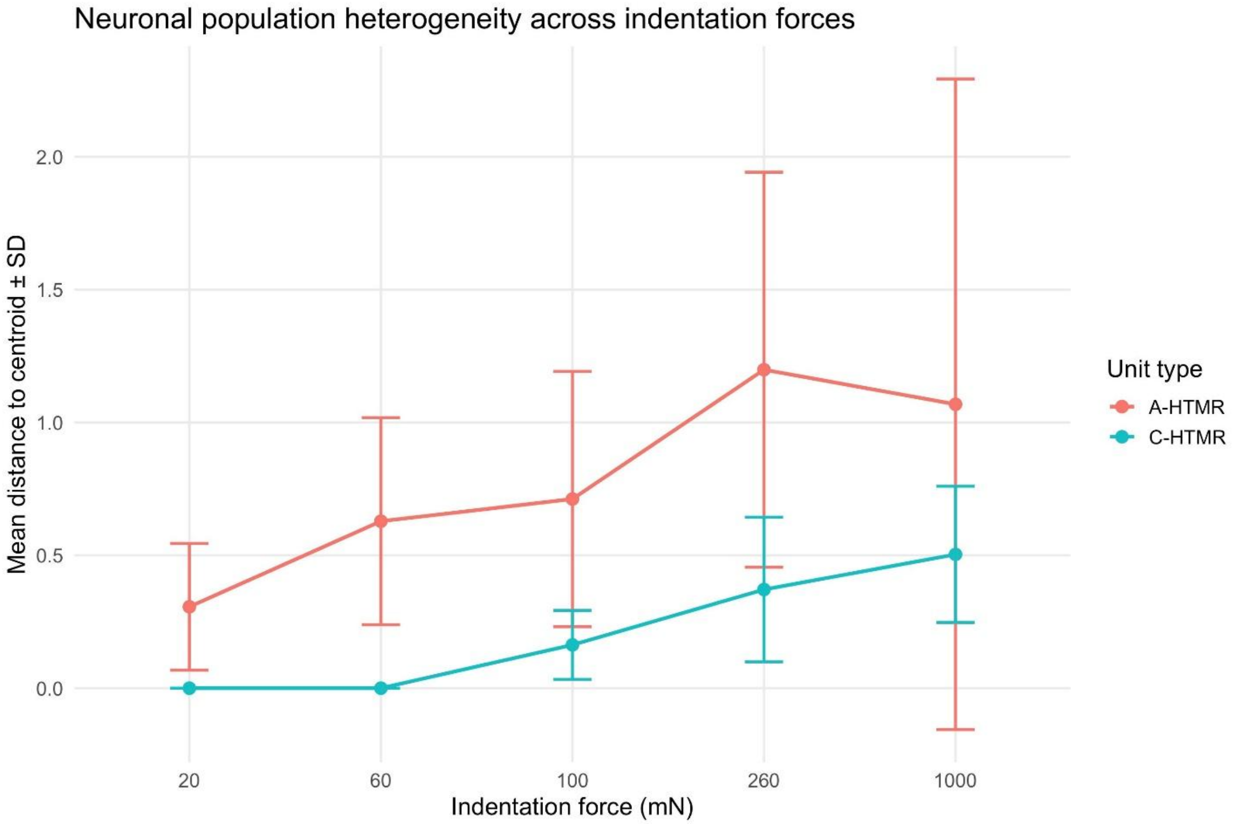

Supplementary Figure 1: Heterogeneity of neuronal populations across indentation forces. A-HTMRs showed greater mean distance to centroid (± SD) than C-HTMRs at each force level, indicating higher response variability. Increasing indentation force was also associated with larger mean distances to centroid (± SD).
